# Echocardiographic Findings in Jordanian Atrial Fibrillation Patients: Analysis from Jo-Fib Study

**DOI:** 10.3390/medicina61020314

**Published:** 2025-02-11

**Authors:** Zaid A. Abdulelah, Kais Al Balbissi, Mohammad Al-Dqour, Ayman Hammoudeh, Ahmed A. Abdulelah

**Affiliations:** 1 Addenbrooke’s Hospital, Cambridge University Hospitals NHS Foundation Trust, Addenbrookes Hospital, Hills Rd., Cambridge CB2 0QQ, UK; 2 School of Medicine, The University of Jordan, Amman 11942, Jordan; kaisalbalbissi@yahoo.com; 3 Quillen College of Medicine, East Tennessee State University, Johnson City, TN 37614, USA; mdqour@gmail.com; 4 Department of Cardiology, Istishari Hospital, Amman 11184, Jordan; a.hammoudeh@istisharihospital.com; 5 Royal Papworth Hospital NHS Foundation Trust, Papworth Rd., Trumpington, Cambridge CB2 0AY, UK; ahmadalhassani99@gmail.com

**Keywords:** atrial fibrillation, anticoagulation, Middle Eastern, echocardiogram

## Abstract

*Background and Objectives:* Atrial fibrillation (AF) carries a huge socioeconomic burden as it is the most encountered cardiac arrhythmia with a significant morbidity. Echocardiographic (Echo) imaging is of monumental value in providing insight into assessing the cardiac function and anatomy, etiology, and risk stratification of AF patients, which will ultimately lead to the best management plan. *Materials and Methods:* A total of 2160 adult patients diagnosed with AF in 18 hospitals and 30 out-patient cardiology clinics in Jordan and 1 hospital in the Palestinian Territories were enrolled in this study from May 2019 to January 2021. Ultimately, 1776 patients were included in the analysis after going through the exclusion criteria. *Results:* The majority of our participants were found to have normal EF at the time of enrollment, with only 31.6% exhibiting a decreased EF. Only 40% of overall patients had Echo evidence of left ventricular hypertrophy (LVH). These patients were older (70.27 ± 10.1 vs. 66.0 ± 14.3, *p* < 0.001), more obese (45.2% vs. 37.3%, *p*-value < 0.001), and had a more frequent occurrence of HTN (89.0% vs. 65.6%, *p* < 0.001) and DM (49.2% vs. 40.1%, *p* < 0.001) when compared to patients without LVH. A proportion of 84.2% of female patients had abnormal left atrial (LA) size (>3.8 cm), in contrast to only 53.4% of males (LA > 4.2 cm). Pulmonary hypertension (PH) was only observed in 27.9% of our patients, and when comparing patients with PH vs. patients without PH, decreased EF (<50%) (36.9% vs. 20.6%, *p* = 0.001), a higher prevalence of OSA (6.7% vs. 3.8%, *p* = 0.009), female predominance (60.3% vs. 39.7%, *p* < 0.001), and older age (70.2 ± 10.7 vs. 66.7 ± 13.6, *p* < 0.001) were observed in patients with PH. *Conclusion:* This study provides the first reported insights on the atrial fibrillation-related echocardiographic findings in a Middle Eastern population. Notably, our study demonstrates that the majority of the studied population have no evidence of LVH and have preserved EF on baseline. However, LA enlargement was extremely frequent among females but not in males, warranting further evaluation to determine the factors contributing to such a difference.

## 1. Introduction

The replacement of the normal sinus rhythm originating from the sinoatrial node by a dispersed and disordered electrical activity results in the characteristic irregularly rapid fibrillatory waves of atrial fibrillation (AF). Among cardiac electrophysiological abnormalities, atrial fibrillation is the most commonly encountered arrhythmia with an age, gender, and race-related prevalence that is approximately less than 1% in individuals younger than 65 years but reaches about 10% in those older than 80 years [1]. White males appear to be most affected, as black individuals and females report a lower prevalence [2]. The prevalence of AF in the general population is approximately 0.95% [3]; however, with the increase in life expectancy, the prevalence is projected to increase in the upcoming years. This is of significance due to the association of AF with significant morbidity and mortality, where the Framingham Study demonstrated that AF is an independent risk factor for cerebrovascular accidents and both overall and cardiac-related mortality [4,5].

In the initial workup of AF, echocardiography is one of the several diagnostic methods that are indicated, with the importance of the utilization of echocardiography in the diagnosis of AF lying in its ability to assess the cardiac structural framework and function to determine the underlying etiology of AF and the risk stratification of AF. Transthoracic echocardiography (TTE) is a reliable tool to assess left atrial (LA) structure, which is of significance as LA size and volume carry predictive values for the subsequent development of AF and prognostic implications. Based on the Framingham Study, the risk of development of AF following a 5 mm increase in LA dimension was around 39% [6]. On the other hand, LA volume is regarded to be more specific than LA size when predicting the prognosis of AF, where it can be utilized following radiofrequency catheter ablation (RFCA) to predict the possibility of AF recurrence [7,8]. With regard to functional assessment, TTE can assess the degree of LA mechanical dysfunction, which is reported to be an independent predictor for the maintenance of sinus rhythm following successful therapeutic interventions [9,10]. Nonetheless, it is important to acknowledge that TTE is associated with limitations such as a poor visualization of thrombi, whether in the left ventricle or the left atrial appendage, and the lesser assessment of valvular diseases in comparison to other modalities of cardiac imaging [11].

Due to the consequential morbidity and mortality of AF, several studies investigating AF in the Middle East Region have been conducted [12,13,14,15,16,17]. However, there remains a lack of consensus on AF in the region. The importance of AF-related studies in the region lies not only in optimizing the approach to AF, but also in exploring the possibility of a deviation from pre-existing findings in the literature due to the presence of a higher prevalence of certain AF risk factors including smoking, diabetes, and obesity in the majority of Middle Eastern communities [18,19,20]. Additionally, studies investigating echocardiographic findings in AF patients in the region are virtually nonexistent, resulting in suboptimal treatment approaches [18,19,20].

Based on the Atrial Fibrillation Registry in Jordan, our study was conducted to determine the transthoracic echocardiographic (TTE) findings in patients with AF, with a comparison of these findings to the reported TTE findings in the literature.

## 2. Materials and Methods

The Jordan Atrial Fibrillation (AF) Study is a prospective, observational, multicenter registry that enrolled 2160 consecutive patients aged >18 years who were diagnosed with AF in 18 hospitals and 30 out-patient cardiology clinics in Jordan and 1 hospital in the Palestinian Territories from May 2019 to February 2021. Data were collected on a standardized clinical study form at the time of enrollment. Additionally, patients were followed-up for one year since the time of enrollment. Baseline data included clinical profiles, cardiovascular risk factors, laboratory data, electrocardiographic (ECG), and echocardiographic features. CHA2DS2-VASc score and HAS BLED scores were calculated for each patient. The utilization of pharmacotherapy including oral anticoagulant medications (OACs) was evaluated at the time of enrollment in this study. The study was conducted in accordance with the declaration of Helsinki while also obtaining informed consents from the enrolled patients. Institutional Review Board (IRB) approval was obtained from the appropriate body.

### 2.1. Echocardiographic Features Studied

The diagnosis of AF was confirmed by (1) a 12-lead ECG; (2) a rhythm strip, lasting >30 s; (3) one or more episodes of AF on the Holter monitor; or (4) a diagnosis by a treating cardiologist. The definitions of AF types, namely first attack of AF, paroxysmal AF, persistent AF, and permanent AF, were according to the American College of Cardiology/American Heart Association/Heart Rhythm Society 2019 update on guidelines for the Treatment of patients with AF [21].

CHA2DS2-VASc score and HAS BLED scores were calculated for each patient according to the 2014 AHA/ACC/HRS Guideline on the management of AF [22].

This study was approved by the Institutional Review Board of each of the participating hospitals and every patient signed a written informed consent. During the study, the patients had adequate information about the study and were informed about the study results. Moreover, patients had the right to withdraw from the study at any time without having to provide an excuse for their decision.

Overall, 384 patients were excluded from this study. Exclusion criteria included inconclusive clinical characteristic data and missing echocardiographic data. Accordingly, 1776 patients were included in this analysis.

### 2.2. Statistical Analysis

All statistical analysis was conducted on the statistical package for social sciences (SPSS) version 25.0 (SPSS Inc., Chicago, IL, USA). Descriptive statistics were performed using the means and standard deviation (SD) to describe the continuous variables and percentages to describe the categorical variables. An independent *t*-test was used to compare means and the Chi-square test was used to compare categorical data. *p* < 0.05 was considered statistically significant.

## 3. Results

A total of 1776 patients were evaluated with regard to baseline characteristics, valvular vs. non-valvular atrial fibrillation (AF), left ventricular hypertrophy, pulmonary hypertension, left atrial size, ejection fraction, and the presence of normal echocardiography.

### 3.1. Baseline Characteristics

Out of the 1776 patients, the mean age was 67.7 ± 13 years old and 53.3% (*n* = 947) of participants were females. Only 27.9% of patients had their first episode of AF at the time of enrollment. Moreover, paroxysmal AF was the most common type of AF in this cohort (35.1%, *n* = 523), followed by permanent AF in 27.8% (*n* = 493) of cases. The proportion of patients who were hospitalized at the time of enrollment was 29.3% (*n* = 521). Further baseline characteristics for the study participants are summarized in Table 1.

### 3.2. Valvular vs. Non-Valvular AF

**Figure 1:**
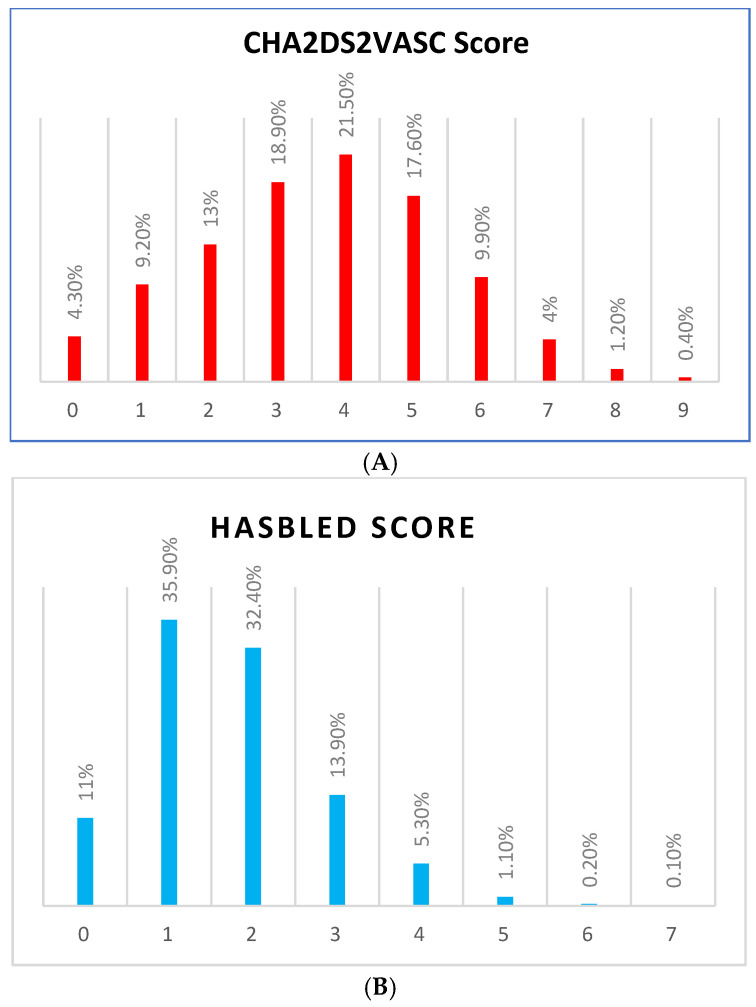
(**A**) The prevalence of different CHA2DS2VASC scores in patients with NVAF. The x-axis presents the score and the y-axis presents the percentage. Non-valvular atrial fibrillation = NVAF. (**B**) The prevalence of different HASBLED scores in patients with NVAF. The x-axis presents the score and the y-axis presents the percentage. Non-valvular atrial fibrillation = NVAF.

A small minority of patients (7.8%, *n* = 139) had valvular AF (VAF), i.e., significant mitral stenosis or a mechanical valve. Patients with non-valvular AF (NVAF) had a mean CHA2DS2–VASc score of 3.67 ± 1.83 and a mean HAS-BLED score of 1.71 ± 1.12. Notably, a high-risk CHA2DS2–VASc score (≥4) was prevalent in 54.6% (*n* = 894) of patients with NVAF, while a low prevalence of a high HAS-BLED score (≥4) occurred in only 6.7% (*n* = 110) of NVAF cases. Detailed descriptions of CHA2DS2–VASc and HAS-BLED scores are summarized in Figure 1A,B.

**Figure 2:**
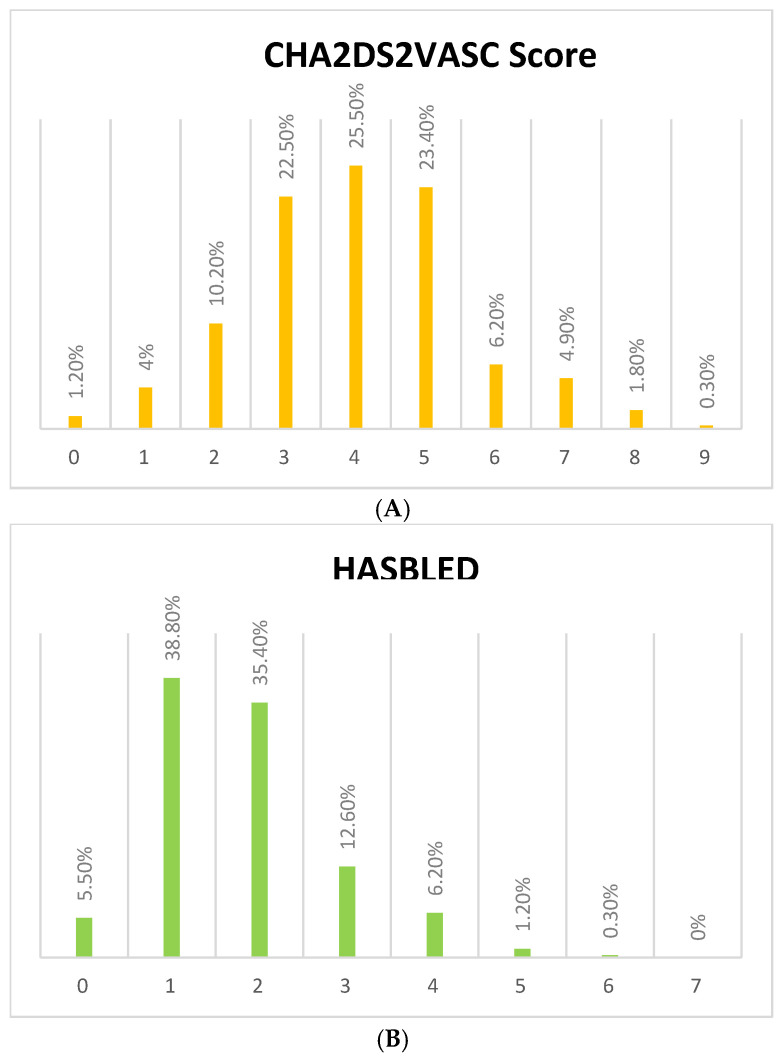
(**A**) The prevalence of different CHA2DS2VASC scores among patients with NVAF and other valvular diseases. The x-axis presents the score and the y-axis presents the percentage. Non-valvular atrial fibrillation = NVAF. (**B**) The prevalence of different HASBLED scores among patients with NVAF and other valvular diseases. The x-axis presents the score and the y-axis presents the percentage. Non-valvular atrial fibrillation = NVAF.

Furthermore, 20.8% (*n* = 370) of all patients enrolled in our study had other valvular disorders, i.e., significant mitral regurgitation, significant aortic stenosis, significant aortic regurgitation, and significant tricuspid regurgitation. Of those patients who had NVAF and other valvular disorders (87.8%, *n* = 325), they had a mean CHA2DS2–VASc score of 4.0 ± 1.58 and a mean HAS-BLED score of 1.8 ± 1.05. Of note, a high-risk CHA2DS2–VASc score (≥4) was prevalent in 62.2% (*n* = 202) of NVAF cases who had other valvular diseases. However, they had a low prevalence of a high HAS-BLED score (≥4), occurring in only 7.7% (*n* = 25) of NVAF cases who had other valvular diseases. Detailed results of CHA2DS2–VASc and HAS-BLED scores are summarized in Figure 2A,B.

When patients with NVAF are compared to their counterparts with VAF, they are more likely to be of older age (68.3 ± 13 years vs. 61.6 ± 12 years, *p*-value < 0.001) and have a higher Body Mass Index (BMI), where 41.3% of NVAF patients were obese (BMI ≥ 30), whereas only 30.6% of VAF patients were obese (*p*-value < 0.001). Additionally, NVAF patients had a higher prevalence of hypertension (HTN) (76.8% vs. 53.2%, *p*-value < 0.001), diabetes (DM) (45.0% vs. 28.8%, *p*-value < 0.001), and dyslipidemia (46.1% vs. 23.7%, *p*-value < 0.001). Further comparisons between NVAF and VAF are discussed in Table 2.

### 3.3. Left Ventricular Hypertrophy

Only 40% (*n* = 711) of patients had echocardiographic evidence of left ventricular hypertrophy (LVH). LVH was defined as an increase in ventricular mass, whether due to increased wall thickening or ventricular dilation [23]. When comparing patients with LVH to patients without LVH, they tend to be of older age (70.27 ± 10.1 years vs. 66.0 ± 14.3 years, *p*-value < 0.001) and more obese (45.2% vs. 37.3%, *p*-value < 0.001). Moreover, patients with LVH have a higher prevalence of HTN (89.0% vs. 65.6%, *p*-value < 0.001), DM (49.2% vs. 40.1%, *p*-value < 0.001), and dyslipidemia (47.4% vs. 42.3%, *p*-value = 0.036). Notably, patients with LVH were more likely to have malignancy (7.0% vs. 4.0%, *p*-value = 0.006) and obstructive sleep apnea (OSA) (6.0% vs. 3.6%, *p*-value = 0.014). However, patients with LVH have a lower prevalence of heart failure (HF) (20.4% vs. 28.34, *p*-value < 0.001). Further comparisons between patients with LVH and patients without LVH are summarized in Table 3.

### 3.4. Pulmonary Hypertension

A minority of patients (27.9%, *n* = 496) had echocardiographic evidence of pulmonary hypertension (PH). Patients with PH when compared to patients without PH are more likely to be of older age (70.2 ± 10.7 years vs. 66.7 ± 13.6 years, *p*-value < 0.001) and to be females (60.3% vs. 39.7%, *p*-value < 0.001). Additionally, patients with PH have a higher prevalence of HF (36.9% vs. 20.6%, *p*-value < 0.001), OSA (6.7% vs. 3.8%, *p*-value = 0.009), and cancer (7.7% vs. 4.3%, *p*-value = 0.004). However, patients with PH are less likely to be obese (36.6% vs. 41.9%, *p*-value = 0.018). Further comparisons between patients with PH and patients without PH are summarized in Table 4.

### 3.5. Left Atrial Size

The mean LA size for all the 1776 patients involved in this study was 4.32 ± 0.78 cm. In females, the mean LA size was 4.34 ± 0.78 cm, with 84.2% of females exhibiting an abnormal LA size of ≥3.8 cm. Moreover, females with abnormal LA size are more likely to be older (70.2 ± 10.7 years vs. 64.0 ± 12.9 years, *p*-value = 0.001) and obese (47.3% vs. 41.4%, *p*-value = 0.038) when compared to their counterparts who had normal LA size. When comparing females with abnormal LA size with those with normal LA size, they have a higher prevalence of HTN (80.8% vs. 72.0%, *p*-value = 0.014) and HF (23.7% vs. 10.7%, *p*-value < 0.001). Further comparisons between females with abnormal LA size and those with normal LA size are summarized in Table 5.

Males had a mean LA size of 4.29 ± 0.79 cm, with only 53.4% of them exhibiting an abnormal LA size of ≥4.2 cm. Additionally, males with abnormal LA size tend to be of older age (69.5 ± 11.5 years vs. 62.0 ± 16.4 years, *p*-value < 0.001) with a higher prevalence of HTN (73.8% vs. 65.5%, *p*-value = 0.009) and HF (39.1% vs. 17.9%, *p*-value < 0.001). Further comparisons between males with abnormal LA size and those with normal LA size are summarized in Table 6.

### 3.6. Ejection Fraction

Mean ejection fraction (EF) was 53.5% ± 12.2, with the majority of patients (68.4%, *n* = 1215) exhibiting a normal EF (>50% [24]). Patients with normal EF were more likely to be younger (67.0 ± 13.3 years vs. 70.0 ± 11.5 years, *p*-value = 0.001) and females (57.6% vs. 42.4%, *p*-value < 0.001) when compared with patients who had decreased EF (<50%). Moreover, patients who had normal EF had a lower prevalence of DM (41.1% vs. 50.6%, *p*-value = 0.001), HF (10.5% vs. 71.4%, *p*-value < 0.001), and malignancy (4.4% vs. 8.0%, *p*-value = 0.004). However, patients with normal EF had a higher prevalence of HTN (76.3% vs. 71.0%, *p*-value = 0.027) and were more likely to be obese (42.6% vs. 33.7%, *p*-value = 0.008). Further data about EF are summarized in Table 7.

### 3.7. Normal Echocardiography

A small minority of patients (15.4%, *n* = 274) had NVAF and normal echocardiography, i.e., normal LA size, normal EF, no LVH, no PH, and no other valvular disorders. Those patients had a mean age of 58 ± 16.5 years and were more likely to be males (69.3%). The most common type of AF was paroxysmal (63.1%) and 40.1% had their first episode of AF at the time of enrollment. Only 33.6% of the participants were inpatients at the time of enrollment with AF morbidity and mortality being the most common cause of admission (14.6%). Further baseline characteristics for those patients are summarized in Table 8.

## 4. Discussion

The effects of atrial fibrillation (AF) on the cardiac anatomical and physiological framework are well established. However, the transthoracic echocardiogram (TTE) is universally recognized for its pivotal role in assessing the cardiac framework, in addition to the fact that risk stratification and prognostic implications associated with the use of TTE in AF patients are of substantial significance in directing the management of AF. Accordingly, and to the best of our knowledge, we conducted the first study in the Middle East, evaluating the TTE findings in Middle Eastern AF patients to establish an overview regarding the leading findings, and thus to provide the foundational basis for any directive towards altering the approach and management of Middle Eastern AF patients.

Initially, it is important to note that the mean age of our study sample (67.7 ± 13 years old) is considerably higher when compared to an AF registry conducted in a Middle Eastern country, Qatar, whose sample’s mean age ranged from 54.9 to 57.5 during the time period of 1991–2010 [14]. Despite our study being based in a different timeline, 2019–2021, the difference in the mean age between the two studies could be partly explained by not only a higher percentage of expatriates living in Qatar compared to Jordan, but also a considerably high prevalence of overweight and obesity with an estimated 70% and 41% of the Qatari population being overweight and obese, respectively [25]. Nonetheless, Jordan also suffers from soaring prevalence rates in terms of obesity [19], and thus there remains doubt regarding the driving cause behind the difference in samples’ mean ages. However, our sample’s mean age is closer to what is reported in the literature of a mean age of 69 years [26]. With regard to our sample’s gender distribution, the surprising majority, 53.3%, were females in contrast to the widely regarded higher male gender prevalence noted in AF (596 per 100,000 in males vs. 373 per 100,000 in females) [27].

Our study demonstrated a similar prevalence of valvular atrial fibrillation (VAF) and non-valvular atrial fibrillation (NVAF) to previously reported figures in the literature, where only 7.8% of our patients had VAF in contrast to 92.2% who had NVAF, demonstrating its consistency to other countries’ findings with a recent study displaying that 9.1% of their sample had VAF and 90.9% had NVAF [28]. Furthermore, our study demonstrated a statistically significant correlation between older age, BMI, DM, and dyslipidemia and NVAF in comparison with VAF, indicating the importance of thorough clinical evaluation for NVAF in such patients. Additionally, this validates our findings due to our results’ consistency with the established evidence in the literature that signifies the correlation between these conditions and NVAF [29,30,31]. Notably, 20.8% (n = 370) of our patients had other valvular diseases at the time of enrollment with a strikingly significant portion of NVAF patients with other valvular diseases (87.8%, n = 325) having a high-risk CHA2DS2–VASc score (≥4) (62.2%, n = 202). Similarly, Vo et al. [28] reported that NVAF patients with other valvular diseases demonstrated the highest CHA2DS2–VASc score in their sample, thus signifying the importance of anticoagulants, with the current recommendations supporting the use of direct oral anticoagulants (DOACs) as an alternative to vitamin K antagonists [28].

The evaluation of LA size in AF patients is of paramount significance due to compelling evidence demonstrating poorer outcomes in patients with AF who have documented LA enlargement, particularly in terms of the likelihood of successful cardioversion and radiofrequency catheter ablation [32,33]. The mean LA size in our sample was 4.32 ± 0.78 cm, with the mean LA size in females and males being 4.34 ± 0.78 and 4.29 ± 0.79 cm, respectively. Accordingly, our study demonstrates a significant portion of the patients exhibiting an abnormal LA size, where 84.2% of females and 53.4% males have TTE-documented LA enlargement. This soaring prevalence of LA enlargement is alarming as it presents an enigma in the management of these patients due to the association of LA enlargement in AF patients with unsatisfactory treatment outcomes as it is considered resistant to treatment, particularly in maintaining sinus rhythm [33]. Accordingly, this further warrants the need of early recognition and management of AF in order to optimize outcomes prior to the development of LA enlargement. Moreover, it is unclear why more females than males in our study population had LA enlargement. It has been previously reported that males tend to have a larger LA size in comparison to females mostly due to the variation in the body size and weight [34]. Therefore, further assessment of the factors leading to this considerable difference needs to be performed. However, one difference leading to this could be the increased obesity rates among females in comparison to males in the Middle East [35].

With regard to pulmonary hypertension (PH), it is important to acknowledge that 27.9% of patients had echocardiographic evidence of PH. In the context of AF and PH, there has been a reported increased risk of morbidity and mortality, in addition to challenges in the management [36]. Therefore, it is important to acknowledge that this subset of patients in our population, who require more specialist input, might experience a high incidence of AF-related complications [36]. This is particularly of significance in the Middle East where pulmonary hypertension specialists are very scarce.

Assessing for LVH in AF is of momentous significance due to its implications and risk towards morbidity and mortality [30]. Our study revealed that only 40% of AF patients had LVH on TTE at the time of enrollment. When compared to the existing literature, there is a considerable variation between studies concerning the proportion of AF patients with LVH, with Hijazi et al. [37] reporting only 23.8% of their sample size exhibiting LVH, while Proietti et al. [38] reported that 56% of their sample had LVH. Nonetheless, it is crucial to mention that Hijazi et al. [37] involved participants from 44 countries, in contrast to Proietti et al. [38], thus indicating the potential likelihood of variation in LVH among AF patients in different geographical locations, in addition to other factors potentially contributing towards heterogeneity in the investigated populations. In regard to the conditions associated with LVH in AF patients, there is compelling evidence correlating the higher prevalence of older age, HTN, and DM among this subgroup of patients [37,38] with our study, demonstrating a similar correlation between AF patients with LVH and older age, HTN, and DM. Consequently, this demonstrates that the correlation of AF with certain diseases and conditions may not be altered by geographical regions. Moreover, our study also revealed a statistically significant correlation between AF patients with LVH and dyslipidemia, obesity, OSA, and malignancy. Surprisingly, however, our study demonstrated a statistically significant (*p*-value < 0.001) correlation between patients with AF and LVH on TTE with a lower prevalence of heart failure, in contrast to the existing findings in the literature [37].

The majority of our participants were found to have normal EF at the time of enrollment, with only 31.6% exhibiting a decreased EF. Interestingly, there is evidence of a decreased impact on mortality by AF in patients with moderate to severe LV dysfunction (EF from 26 to 40% for moderate, and ≤25% for severe) and it is regarded to not be a predicator of mortality in these two subgroups [39]. As a consequence, the evaluation and documentation of EF in AF patients could be of prognostic significance and, accordingly, aid in risk stratification due to the presented evidence. Moreover, the presence of normal EF and the absence of LVH, as encountered with the majority of our studied population, significantly impact treatment modalities and strategies as they might reduce the need for anticoagulation in the absence of other risk factors and allow for attempting pharmacological treatment modalities at the first instance in view of preserved heart function and structure. Thus, our findings provide the foundational basis for tailored AF treatment strategies in the Middle East.

Intriguingly, 15.4% of our participants (n = 274) who were diagnosed with NVAF revealed normal TTE at the time of enrollment, with a mean age of 58 ± 16.5 years and the majority of the participants, 69.3%, being males. The presence of normal TTE in patients with AF in the absence of any other cardiac diseases is commonly referred to as lone AF [40,41] despite the presence of recommendations to avoid the use of this term due to a wide variation in the definition of lone AF [42]. Due to the variation in the definition, and thus the utilized diagnostic tools, the prevalence of lone AF is highly variable, ranging from as low as 0.2% to up to 68% [42,43]. With regard to the prognosis in patients with lone AF, there are no clear findings, due to contradicting results in the literature, which are proposed to be a consequence of the heterogenicity in the definition of lone AF [42].

Despite the significant findings of this study as evidenced by its correlation to the findings of other studies investigating the echocardiographic findings in AF among different populations, this study is limited in certain aspects. Initially, our study involved evaluating TTE findings in AF only, without any evaluation of transesophageal echocardiographic (TEE) findings in the study evaluation which would ultimately provide a substantially more comprehensive clinical profile. However, it is noteworthy that TEE is usually not indicated in all patients and thus was not considered to be part of the study. Additionally, despite the fact that Middle Eastern populations share a significant number of factors and conditions, there remains some differences, particularly the prevalence of certain diseases among different Middle Eastern countries and different healthcare disparities across the region, that potentially limit the extrapolation of the data to the entire Middle Eastern population. Nonetheless, we adamantly believe that our findings provide valuable insight regarding the TTE findings in AF Middle Eastern populations, especially since no prior reported studies evaluated this population, while also igniting the need for studies evaluating the findings in other Middle Eastern countries.

## 5. Conclusions

In conclusion, our study demonstrates the echocardiographic findings in a Middle Eastern population in comparison to TTE findings in Western populations, thus further emphasizing the vitality of utilizing TTE in AF patients for its diagnostic and prognostic implications. Notably, our study demonstrated that the majority of the studied population have preserved LV function and an absence of LVH, all of which are factors contributing to a higher response rate to pharmacological and ablation treatment modalities for the AF. Nonetheless, a multi-national Middle Eastern study is warranted to further explore potential differences between the different countries in the region, as well as the influence of different regional lifestyle factors.

## Figures and Tables

**Table 1:** The prevalence of different co-morbidities of the study sample at the time of enrollment. Number = N, atrial fibrillation = AF, Hypertension = HTN, Diabetes Mellitus = DM, Coronary Artery Disease = CAD, Heart Failure = HF.

Variable	Category	N (%)
**Age**		67.7 ± 13 Years
**Sex**	Male	827 (46.6%)
	Female	947 (53.4%)
**HTN**	No	444 (25%)
	Yes	1332 (75%)
**DM**	No	999 (56.3%)
	Yes	777 (43.8%)
**Dyslipidmia**	No	988 (55.6%)
	Yes	788 (44.4%)
**Admission Type**	Out Patient	1255 (70.7%)
	In Patient	521 (29.3%)
**First Episode of AF**	No	1281 (72.1%)
	Yes	495 (27.9%)
**Type of AF**	Paroxysmal	623 (35.1%)
	Presistent	282 (15.9%)
	Long Standing	377 (21.2%)
	Permanent	493 (27.8%)
**Valvular Disease**	No	1620 (91.2%)
	Yes	156 (8.8%)
**CAD**	No	1591 (89.6%)
	Yes	185 (10.4%)
**HF**	No	1329 (74.8%)
	Yes	447 (25.2)
**Sleep Apnea**	No	1695 (95.4%)
	Yes	81 (4.6%)
**Cancer**	No	1683 (94.8%)
	Yes	93 (5.2%)

**Table 2:** Comparison between NVAF and VAF patients. Number = N, Hypertension = HTN, Diabetes Mellitus = DM, Coronary Artery Disease = CAD, Heart Failure = HF, non-valvular atrial fibrillation = NVAF, valvular atrial fibrillation = VAF, Chronic Kidney Disease = CKD, Body Mass Index = BMI. An independent *t*-test was used to compare means and the Chi-square test was used to compare categorical data. *p* < 0.05 was considered statistically significant.

Variable	Category	*n* (%)	VAF (%)	NVAF (%)	*p* -Value
**N**	Total	1776 (100%)	139 (7.8)	1637 (92.2%)	
**Age**		67.7 ± 13	68.3 ± 13	61.6 ± 12	<0.001
**Gender**					
	Male	827 (46.6%)	45(32.2%)	782 (47.8%)	<0.001
	Female	947 (53.4%)	94 (67.6%)	853 (52.2%)	
**HTN**		1332 (75%)	74 (53.2%)	1258 (76.8%)	<0.001
**DM**		777 (43.8%)	40 (28.8%)	737 (45%)	<0.001
**Dyslipidemia**		788 (44.4%)	33 (23.7%)	755 (46.1%)	<0.001
**Sleep Apnea**		81 (4.6%)	3 (2.2%)	78 (4.8%)	0.157
**CAD**		185 (10.4%)	10 (7.2%)	175 (10.7%)	0.195
**HF**		447 (25.2%)	27 (19.4%)	420 (25.7%)	0.729
**Thyroid Disorder**		189 (10.6%)	16 (11.5%)	173 (10.6%)	0.729
**CKD**		174 (9.8%)	9 (6.6%)	165 (10.1%)	0.17
**BMI ≥ 30**		651 (40.5%)	38 (30.6%)	613 (41.3%)	<0.001

**Table 3:** Comparison between patients with and without LVH. Number = N, Left Ventricular Hypertrophy = LVH, Hypertension = HTN, Diabetes Mellitus = DM, Coronary Artery Disease = CAD, Heart Failure = HF, Chronic Kidney Disease = CKD, Body Mass Index = BMI. An independent *t*-test was used to compare means and the Chi-square test was used to compare categorical data. *p* < 0.05 was considered statistically significant.

Variable	Category	N (%)	No LVH (%)	LVH (%)	*p* -Value
**N**	Total	1776 (100%)	710 (40%)	1064 (60%)	
**Age**		67.7 ± 13	66 ± 14.3	70.3 ± 10.1	<0.001
**Gender**					0.497
	Male	827 (46.6%)	503 (47.3%)	324 (45.6%)	
	Female	947 (53.4%)	561 (52.7%)	386 (54.4%)	
**HTN**		1332 (75%)	699 (65.6%)	633 (89%)	<0.001
**DM**		777 (43.8%)	427 (40.1%)	350 (49.2%)	<0.001
**Dyslipidemia**		788 (44.4%)	451 (42.3%)	337 (47.4%)	0.036
**Sleep Apnea**		81 (4.6%)	38 (3.6%)	43 (6%)	0.014
**CAD**		185 (10.4%)	130 (12.2%)	55 (7.7%)	0.003
**HF**		447 (25.2%)	302 (28.4%)	566 (20.4%)	<0.001
**Thyroid Disorder**		189 (10.6%)	117 (11%)	72 (10.1%)	0.565
**CKD**		174 (9.8%)	105 (9.9%)	69 (9.7%)	0.915
**BMI ≥ 30**		651 (40.5%)	363 (37.3%)	288 (45.2%)	<0.001

**Table 4:** Comparison between patients with and without PH. Number = N, pulmonary hypertension = PHTN Hypertension = HTN, Diabetes Mellitus = DM, Coronary Artery Disease = CAD, Heart Failure = HF, Chronic Kidney Disease = CKD, Body Mass Index = BMI. An independent *t*-test was used to compare means and the Chi-square test was used to compare categorical data. *p* < 0.05 was considered statistically significant.

Variable	Category	N (%)	No PHTN (%)	PHTN (%)	*p* -Value
**N**	Total	1776 (100%)	1280(72.1%)	496(27.9%)	
**Age**		67.7 ± 13	66.8 ± 13.6	70.2 ± 10.7	<0.001
**Gender**					<0.001
	Male	827 (46.6%)	630 (49.3%)	197 (39.7%)	
	Female	947 (53.4%)	648 (50.7%)	299 (60.3%)	
**HTN**		1332 (75%)	950 (74.2%)	382 (77%)	0.222
**DM**		777 (43.8%)	554 (43.3%)	223 (45%)	0.522
**Dyslipidemia**		788 (44.4%)	614 (48%)	174 (35.1%)	<0.001
**Sleep Apnea**		81 (4.6%)	48 (3.8%)	33 (6.7%)	0.009
**CAD**		185 (10.4%)	136 (10.6%)	49 (9.9%)	0.644
**HF**		447 (25.2%)	264 (20.6%)	183 (36.9%)	<0.001
**Thyroid Disorder**		189 (10.6%)	125 (9.8%)	64 (12.9%)	0.054
**CKD**		174 (9.8%)	108 (8.4%)	66 (13.3%)	0.002
**BMI ≥ 30**		651 (40.5%)	490 (41.9%)	161 (36.9%)	0.018

**Table 5:** Comparison of female patients with and without abnormal LA size. Number = N, Left Atrium = LA, Hypertension = HTN, Diabetes Mellitus = DM, Coronary Artery Disease = CAD, Heart Failure = HF, Chronic Kidney Disease = CKD, Body Mass Index = BMI. An independent *t*-test was used to compare means and the Chi-square test was used to compare categorical data. *p* < 0.05 was considered statistically significant.

Variable	Category	N (%)	Normal LA (%)	Abnormal LA (%)	*p* -Value
**N**	Total	947 (100%)	150 (15.8%)	797 (84.2%)	
**Age**			64.0 ± 12.9	70.2 ± 10.7	0.001
**HTN**		752 (79.4%)	108 (72%)	644 (80.8%)	0.014
**DM**		436 (46%)	59 (39.33%)	377 (47.3%)	0.072
**Dyslipidemia**		424 (44.4%)	70 (46.7%)	453 (44.4%)	0.611
**Sleep Apnea**		46 (4.9%)	5 (3.3%)	41 (5.1%)	0.344
**CAD**		68 (7.2%)	13 (8.7%)	55 (6.9%)	0.442
**HF**		205 (21.6%)	16 (10.7%)	189 (23.7%)	<0.001
**Thyroid Disorder**		138 (14.6%)	21 (914%)	117 (14.7%)	0.829
**CKD**		76 (8.0%)	7 (4.7%)	69 (8.7%)	0.099
**BMI ≥ 30**		393 (46.3%)	54 (40.6%)	339 (47.3%)	0.038

**Table 6:** Comparison of male patients with and without abnormal LA size. Number = N, Left Atrium = LA, Hypertension = HTN, Diabetes Mellitus = DM, Coronary Artery Disease = CAD, Heart Failure = HF, Chronic Kidney Disease = CKD, Body Mass Index = BMI. An independent *t*-test was used to compare means and the Chi-square test was used to compare categorical data. *p* < 0.05 was considered statistically significant.

Variable	Category	N (%)	Normal LA (%)	Abnormal LA (%)	*p* -Value
**N**	Total	827 (100%)	385 (46.6%)	442 (53.42%)	
**Age**			62.0 ± 16.4	69.5 ± 11.5	<0.001
**HTN**		578 (69.9%)	252 (65.5%)	326 (73.8%)	0.009
**DM**		339 (41%)	148 (38.4%)	191 (43.2%)	0.164
**Dyslipidemia**		363 (43.9%)	171 (44.4%)	192 (43.4%)	0.778
**Sleep Apnea**		35 (4.2%)	15 (3.9%)	20 (4.5%)	0.654
**CAD**		117 (14.1%)	64 (16.6%)	53 (12%)	0.057
**HF**		242 (29.3%)	69 (17.9%)	173 (39.1%)	<0.001
**Thyroid Disorder**		51 (6.3%)	12 (3.1%)	39 (8.8%)	0.001
**CKD**		98 (11.9%)	33 (8.6%)	65 (14.7%)	0.006
**BMI ≥ 30**		256 (33.8%)	116 (32.8%)	140 (34.7%)	0.849

**Table 7:** The EF of the study participants in correlation with various co-morbidities. Number = N, Ejection Fraction = EF, Hypertension = HTN, Diabetes Mellitus = DM, Coronary Artery Disease = CAD, Heart Failure = HF, Chronic Kidney Disease = CKD, Body Mass Index = BMI. An independent *t*-test was used to compare means and the Chi-square test was used to compare categorical data. *p* < 0.05 was considered statistically significant.

Variable	Category	N (%)	Reduced EF (%)	Normal EF (%)	*p* -Value
**N**	Total	1776 (100%)	561 (31.6%)	1213 (68.4%)	
**Age**		67.7 ± 13	69.4 ± 11.7	66.9 ± 13.5	<0.001
**Gender**					<0.001
	Male	827 (46.6%)	310 (37.5%%)	517 (62.5%%)	
	Female	947 (53.4%)	215 (26.5%)	696(29.1%)	
**HTN**		1332 (75%)	413 (31%)	919 (69%)	0.361
**DM**		777 (43.8%)	217 (35.3%)	503(64.7%)	0.003
**Dyslipidemia**		788 (44.4%)	270 (34.3%)	518 (65.7%)	0.03
**Sleep Apnea**		81 (4.6%)	28 (34.6%)	53 (65.4%)	0.555
**CAD**		185 (10.4%)	77 (41.6%)	108 (58.4%)	0.002
**HF**		447 (25.2%)	330 (73.8%)	117 (26.2%)	<0.001
**Thyroid Disorder**		189 (10.6%)	56 (29.6%)	133 (70.4%)	0.54
**CKD**		174 (9.8%)	69 (39.7%)	105 (60.3%)	0.016
**BMI ≥ 30**		651 (40.5%)	189 (29%)	462 (71%)	0.148

**Table 8:** The characteristics of patients with normal echocardiography. Number = N, atrial fibrillation = AF, Hypertension = HTN, Diabetes Mellitus = DM, Coronary Artery Disease = CAD, Heart Failure = HF.

Variable	Category	N (%)
**Age**		58.4 ± 16.5 Years
**Sex**	Male	190 (69.6%)
	Female	83 (30.4%)
**HTN**	No	113 (41.2%)
	Yes	161 (58.8%)
**DM**	No	188 (68.6%)
	Yes	86 (31.4%)
**Dyslipidmia**	No	153 (55.8%)
	Yes	121 (44.2%)
**First Episode of AF**	No	164 (59.9%)
	Yes	110 (40.1%)
**Type of AF**	Paroxysmal	173 (63.1%)
	Presistent	27 (9.9%)
	Long Standing	26 (9.5%)
	Permanent	48 (11.7%)
**CAD**	No	238 (86.9%)
	Yes	36 (13.1%)
**HF**	No	265 (96.7%)
	Yes	9 (3.3%)
**Sleep Apnea**	No	266 (97.1%)
	Yes	8 (2.9%)
**Cancer**	No	263 (96%)
	Yes	11 (4%)
**BMI ≥ 30**	No	180 (65.7%)
	Yes	94 (34.3%)

## Data Availability

The raw data supporting the conclusions of this article will be made available by the authors on request.
